# Supplementary material for: Predicting visual function by interpreting a neuronal wiring diagram
Source: Nature. 2024 Oct 2;634(8032):113–23. doi: 10.1038/s41586-024-07953-5 (PMC11446822; doi:10.1038/s41586-024-07953-5)
Supplement: Supplementary file 6 — CRF and ERF predictions for individual TmY4 and TmY9 cells. Analogous to Supplementary Data 3, but for TmY target types. Shown are the top four monosynaptic pathways, the strongest pathway passing through each of the top ten intermediary types (ranking from Extended Data Fig. 7), and the trisynaptic pathway Tm1–TmY–Dm3–TmY (see the section entitled Prediction of spatial normalization). [file 41586_2024_7953_MOESM6_ESM.zip › DataS4/TmY9q/720575940627539786.pdf]

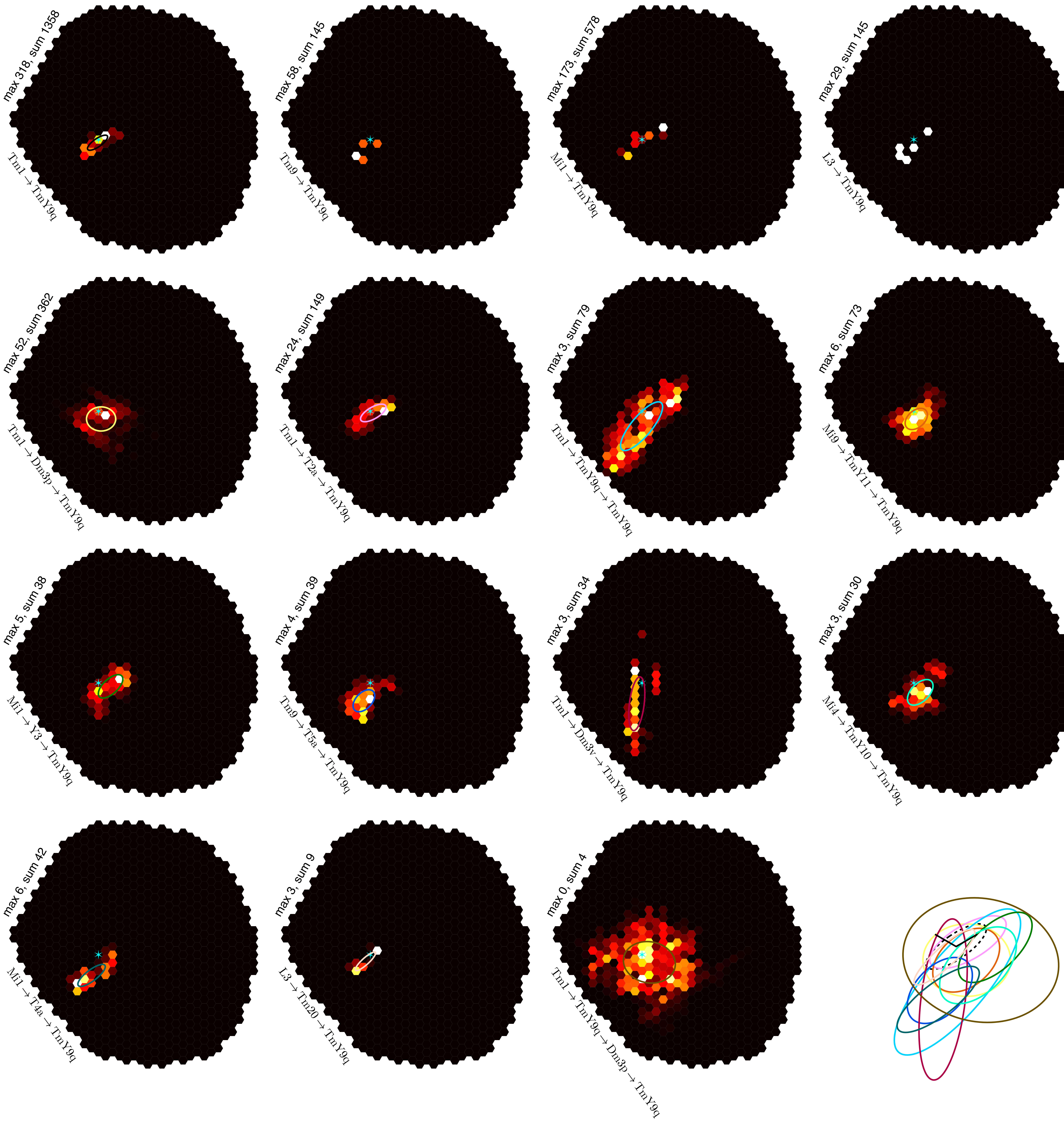
max 318, sum 1358
Tm1 → TmY9q
max 58, sum 145
Tm9 → TmY9q
max 173, sum 578
Mi1 → TmY9q
max 29, sum 145
L3 → TmY9q
max 52, sum 362
Tm1 → Dm3p → TmY9q
max 24, sum 149
Tm1 → T2a → TmY9q
max 3, sum 79
Tm1 → TmY9q → TmY9q
max 6, sum 73
Mi9 → TmY11 → TmY9q
max 5, sum 38
Mi1 → Y3 → TmY9q
max 4, sum 39
Tm9 → T5a → TmY9q
max 3, sum 34
Tm1 → Dm3v → TmY9q
max 3, sum 30
Mi4 → TmY10 → TmY9q
max 6, sum 42
Mi1 → T4a → TmY9q
max 3, sum 9
L3 → Tm20 → TmY9q
max 0, sum 4
Tm1 → TmY9q → Dm3p → TmY9q
